# Adaptive Diversity of Beech Seedlings Under Climate Change Scenarios

**DOI:** 10.3389/fpls.2018.01918

**Published:** 2019-01-08

**Authors:** Georgios Varsamis, Aristotelis C. Papageorgiou, Theodora Merou, Ioannis Takos, Chrisovalantis Malesios, Apostolos Manolis, Ioannis Tsiripidis, Oliver Gailing

**Affiliations:** ^1^Forest Genetics Laboratory, Department of Forestry, Environmental Management and Natural Resources, Democritus University of Thrace, Orestiada, Greece; ^2^Department of Forestry and Natural Environment Management, Eastern Macedonia and Thrace Institute of Technology, Drama, Greece; ^3^Department of Agricultural Development, Democritus University of Thrace, Orestiada, Greece; ^4^Department of Botany, School of Biology, Aristotle University of Thessaloniki, Thessaloniki, Greece; ^5^Department of Forest Genetics and Forest Tree Breeding, Faculty of Forest Sciences and Forest Ecology, Georg-August University of Göttingen, Göttingen, Germany

**Keywords:** *Fagus* sp., phenology, survival, height, adaptation, common garden experiment

## Abstract

The ability of beech (*Fagus sylvatica* L.) populations to adapt to the ongoing climate change is especially important in the southern part of Europe, where environmental change is expected to be more intense. In this study, we tested the existing adaptive potential of eight beech populations from two provenances in N.E. Greece (Evros and Drama) that show differences in their environmental conditions and biogeographical background. Seedling survival, growth and leaf phenological traits were selected as adaptive traits and were measured under simulated controlled climate change conditions in a growth chamber. Seedling survival was also tested under current conditions in the field. In the growth chamber, simulated conditions of temperature and precipitation for the year 2050 were applied for 3 years, under two different irrigation schemes, where the same amount of water was distributed either frequently (once every week) or non-frequently (once in 20 days). The results showed that beech seedlings were generally able to survive under climate change conditions and showed adaptive differences among provenances and populations. Furthermore, changes in the duration of the growing season of seedlings were recorded in the growth chamber, allowing them to avoid environmental stress and high selection pressure. Differences were observed between populations and provenances in terms of temporal distribution patterns of precipitation and temperature, rather than the average annual or monthly values of these measures. Additionally, different adaptive strategies appeared among beech seedlings when the same amount of water was distributed differently within each month. This indicates that the physiological response mechanisms of beech individuals are very complex and depend on several interacting parameters. For this reason, the choice of beech provenances for translocation and use in afforestation or reforestation projects should consider the small scale ecotypic diversity of the species and view multiple environmental and climatic parameters in connection to each other.

## Introduction

The European beech (*Fagus sylvatica* L.) is one of the most important tree species in the continent, both economically and ecologically. Its geographical range extends from Scandinavia to the Mediterranean covering various habitats (Bolte et al., 2007; Willner et al., 2017). It is generally considered as an oceanic species that can grow in areas with mild winters and moist summers, sensitive to intense drought periods in the growing season (Fotelli et al., 2001; Leuschner et al., 2001; Bolte et al., 2007; Granier et al., 2007; Pšidová et al., 2015). Several studies report that beech populations in Southern Europe have faced strong selective pressures during the last decades (Jump et al., 2006; Piovesan et al., 2008), which are expected to become more intense because of future changes in rainfall patterns and temperatures under the forthcoming climate change (Charru et al., 2010; Rita et al., 2014), arising concerns about the survival dynamics of the European populations (Bréda et al., 2006; Geßler et al., 2007). Besides increased drought, climate change is expected to cause temporal shifts in the occurrence of cold events, too early or too late in respect to the winter period. To overcome these negative consequences on the future performance of beech forests in afforestation programs in Europe, several authors suggest testing and using beech ecotypes that are adapted to a less oceanic climate (Schraml and Rennenberg, 2002; Nielsen and Jørgensen, 2003; Meier and Leuschner, 2008; Rose et al., 2009), such as the refugial beech populations from Southern Europe (Rennenberg et al., 2004; Geßler et al., 2007) that are expected to be adapted both to cold events and extended periods of drought during their growing season (St Clair and Howe, 2007; Fotelli et al., 2009; Eilmann et al., 2014; Thiel et al., 2014; Dounavi et al., 2016).

The adaptive potential of tree populations can be described through various parameters such as growth, survival and shifts in phenology (Eckhart et al., 2017). Seedling growth can be severely affected by abiotic stressors such as temperature and water deficiency. As climate becomes warmer and summer precipitation is expected to decline, beech populations may face intense drought periods (Geßler et al., 2007). Under water stress, plants usually decrease growth in terms of both height and biomass accumulation because of minimization in carbon fixation through photosynthesis. In addition, low soil water potential affects hydraulic traits (e.g., conductivity) and can create xylem cavities leading to plant mortality (Bolte et al., 2016). For this reason, survival under environmental stress is an important adaptive trait, since it reflects the regeneration dynamics of populations (Ngulube, 1989; Sexton et al., 2002; Matías and Jump, 2014).

Leaf phenology is a key adaptive trait that determines carbon balance (production and accumulation) and the overall growth of plant species, while also affecting ecosystem productivity (Kramer et al., 2000; Larcher, 2003). Bud burst, and leaf senescence are the most important leaf phenological traits used in studies, since they mark the onset, duration and ending of a species growth period. Bud burst reflects the transition phase from the winter dormancy to the onset of next year's growth period and requires a preceding chilling period (Heide, 1993; Kramer et al., 2017). Late bud burst can protect trees from late frosts but can also shorten their growth period (Lechowicz, 1984; Višnjić and Dohrenbusch, 2004). Bud burst is referred to be under genetic-provenance control (Robson et al., 2011, 2018), while it is can be also directly affected by environmental factors such as temperature and photoperiod (e.g., Heide, 1993; Yan and Wallace, 1996; Basler and Körner, 2014; Schüler and Liesebach, 2015; Kramer et al., 2017). However, a limited number of studies exist on the possible effect of water availability on bud burst timing of temperate forest trees (e.g., Morin et al., 2010; Kuster et al., 2014). The time of leaf senescence determines the end of the growing period and the onset of winter dormancy and strongly depends on the environmental factors during the current year. For example, premature leaf senescence can be observed under low summer and autumn precipitation, to mobilize leaf nutrients (Sedigheh et al., 2011; Chen et al., 2015; Gill et al., 2015; Tombesi et al., 2015), while leaf senescence can be delayed by higher autumnal temperatures (Fu et al., 2017), as well as by an increased photoperiod (Way and Montgomery, 2014; Gill et al., 2015). Furthermore, it is also influenced by spring leaf phenology (Fu et al., 2014; Keenan and Richardson, 2015; Panchen et al., 2015) and at the same time it can affect leaf flushing in the next year (Heide, 2003).

Since the overall response of plants to abiotic and biotic stressors is determined by both environmental and genetic factors that act in combination, common garden experiments are needed in order to separate environmental from genetic effects on plant adaptive traits and to describe their interactions (Scheepens et al., 2010; Malyshev et al., 2014; de Villemereuil et al., 2016). In addition, provenance tests may contribute to the selection of suitable sources of reproductive material for future forest restoration and management activities (Bezděčková and Matějka, 2015; Carón et al., 2015). Several provenance tests exist in Europe for beech, under field or glasshouse conditions (e.g. von Wühlisch et al., 1995; Nielsen and Jørgensen, 2003; Czajkowski and Bolte, 2006; Gömöry and Paule, 2011; Kreyling et al., 2012; Liesebach, 2012; Schüler et al., 2012; Thiel et al., 2014; Harter et al., 2015; Dounavi et al., 2016; Petkova et al., 2017; Robson et al., 2018). Despite the possible adaptive importance of Greek beech populations for European forestry, no common garden experiments have been established for this species in the country so far. Furthermore, no provenance test in Europe has so far evaluated the adaptive response of beech provenances to expected rainfall distribution patterns under future climate change scenarios.

The aim of our study was to describe the adaptive potential of beech populations in the southeastern part of Europe (N.E. Greece) to climate change. Two hypotheses about the adaptive potential of beech were tested: (a) Beech provenances from sites with longer drought intervals in summer should be better adapted to the expected environmental conditions under climate change and (b) differences in temporal distribution patterns of precipitation should trigger different physiological responses in beech trees. In order to test these hypotheses, a provenance test was established in a growth chamber, where controlled climate change conditions of temperature and precipitation for the year 2050 were simulated. Seedling survival, growth, and leaf phenology were used as adaptive traits for 3 years. Seedling survival was also tested for the same provenances in a field trial under natural conditions allowing comparisons between the adaptability of provenances under both current environmental conditions and the ones anticipated under climate change.

## Materials and Methods

### Area of the Study and Selection of Populations

Northeastern Greece is a mountainous region, with a topographically diverse landscape covering various altitudes. Mt. Rodopi, a long massif shared between Greece and Bulgaria, dominates this region and extends from the east to the west. In the lowlands, the climate is subhumid and submediterranean, with harsh winters and dry summers, while it becomes more humid and temperate with harsh winters and no summer drought in higher altitudes (Mavromatis, 1980). Beech forests are present all over this region, covering habitats with a large variety of environmental conditions (Bergmeier and Dimopoulos, 2001; Tsiripidis et al., 2007). According to genetic and ecological studies, beech populations in N.E. Greece have a complex biogeographic background, since they represent multiple postglacial lineages, originating from different glacial refugia (Hatziskakis et al., 2009; Papageorgiou et al., 2014). At the same time, this region is suggested to be part of a possible introgression zone between two beech species *F. sylvatica* and *F. orientalis* (Gömöry et al., 1999; Hatziskakis et al., 2009, 2011; Kandemir and Kaya, 2009; Papageorgiou et al., 2014; Houston Durrant et al., 2016), with the former species being present in the western part of the region (west Mt. Rodopi, Mt. Falakro, Mt. Menikio) and the latter characterizing the eastern part of the Mt. Rodopi (Christensen, 1997; Tsiripidis and Athanasiadis, 2003; Papageorgiou et al., 2008).

**Figure 1:**
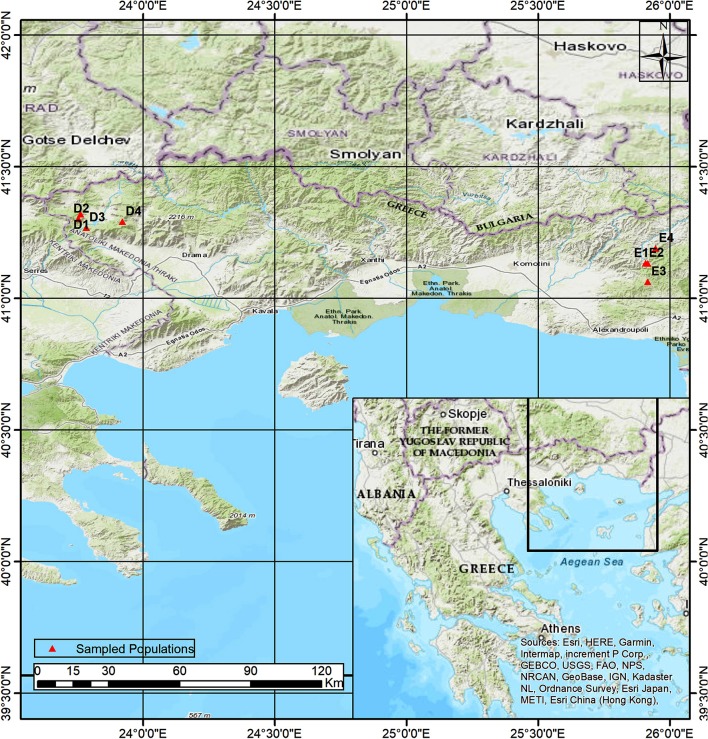
Map of the area of study.

**Figure 2:**
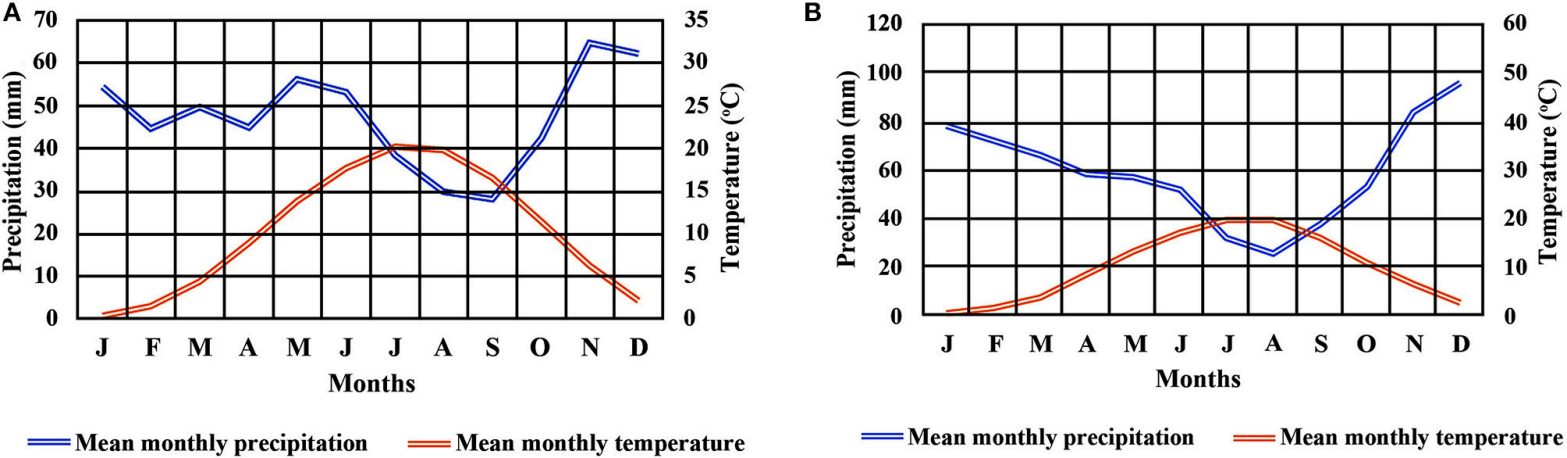
Climate diagram for provenances Drama **(A)** and Evros **(B)**.

Two beech provenances were chosen in N.E. Greece, “Evros,” on the eastern side and “Drama” on the western side of the study area (Figure 1 and Supplement Table 1). Four populations were selected in each provenance (E1–E4 and D1–D4 respectively), representing different postglacial lineages, based on genetic studies (Hatziskakis et al., 2009; Papageorgiou et al., 2014). Due to the absence of long term meteorological data from the area of the selected populations, we used current climatic data available from worldclim.org in a 30-acr seconds resolution (version 1.4) (Hijmans et al., 2005; Souto et al., 2009). Four basic climatic and 19 bioclimatic variables were extracted for the coordinates of each sampled seed parent and the average values were used to describe each population (Supplement Tables 1, 3 and Figures 2A,B). Provenance Evros represents a climatic environment with moist and cold winters, warm and dry summers with an intense (but not long) drought period. The climate in Drama provenance appears to be more continental with relatively moist and more severe winters as well as warm summers with less intense dry periods (Gouvas and Sakellariou, 2011).

### Seed Collection and Germination

Seed collection occurred in autumn 2012, a non-mast production year for beech in the study area, since <50% of the trees produced nuts. In each population, 60–80 seeds per seed tree were collected from 30 trees, totaling 240 families. We consider seeds and later seedlings originating from the same seed tree as a half-sib family. After their transfer to the laboratory, seeds were air-blown to remove the empty ones. The remaining seeds were immersed for 2 min in 35% H_2_O_2_ (Anand and Chanway, 2013) for disinfection and thoroughly rinsed with tap water for several minutes. After the cleaning procedure, seeds were subjected to cold stratification for 90 days (Baskin and Baskin, 2001) at 0°C in petri dishes filled with sterilized sand. Each dish contained 10 randomly selected seeds per family, totaling 300 seeds per population and 1,200 seeds per provenance. Germination was completed during the stratification stage. Seeds that germinated were transferred to plastic pots filled with turf, sand and perlite in a ratio of 4:2:1, respectively, for further development and evaluation. The emerging seedlings were evaluated as normal or abnormal according to ISTA (2018) specifications. Only normal seedlings were included in the following experiments.

### Common Environment Test in a Growth Chamber

In March 2013, the surviving containerized normal seedlings of both provenances were put in a growth chamber under simulated temperature and precipitation levels estimated from the CSIRO MK3 CGM model, according to the expected conditions in the year 2050 (downloaded from Climong.org) (Kriticos et al., 2011) (Supplement Table 2). The specific model was selected for its relevance with the summer drought periods in the Mediterranean region (Marcos and Tsimplis, 2008; Syktus et al., 2011; Ziv et al., 2013; Pulvento et al., 2015). The reference area for the climate simulation in the growth chamber was the location “Agios Georgios” (Drama, Greece) that corresponds to population D4 in this study. Climate change scenario A1 and storyline A1B were selected, with the assumption that the industrial development in the areas of the populations of this study will remain minimal and that there will be a balanced use of all energy sources until 2050 (IPCC, 2014). This model incorporates the indirect effects of greenhouse gases in the change of the estimated future bioclimatic parameters (Kriticos et al., 2011).

For each month, the estimated maximum, minimum and mean temperatures, as well as the precipitation data were extracted from the above dataset (CSIRO MK3 CGM) using the DIVA-GIS software (www.diva-gis.org) (Supplement Table 2). Using these values, a different temperature schedule (change of daily values at 3-h intervals) for each month was created and applied in the growth chamber. Each separate monthly schedule was stable for all the days of each respective month. Light intensity during the day inside the chamber was set according to *in situ* observations that took place in the reference location under clear sky. Annual height growth between the experimental years 2014-2015 and 2015-2016 was also calculated. Light intensity values inside the chamber were set to a range between 0.010 and 0.025 μmol x s^−1^ following the course of natural daylight. The lengths of day and night for each month were adjusted to those under natural conditions in the reference area.

The simulated monthly precipitation height (mm) was converted into water volume (ml) to regulate seedling irrigation, following Brouwer et al. (1985). Besides precipitation height, we tested the effect of precipitation distribution within a month on seedlings, especially during the summer period, since climate change is expected to destabilize the current precipitation frequency and intensity and cause longer drought periods, as well as climate extremes in the Eastern Mediterranean region (Alpert et al., 2002; Gao et al., 2006; Lelieveld et al., 2012). For this reason, precipitation was distributed within each month according to two different irrigation schemes:

1. Irrigation scheme A1 (non-frequent): irrigation of seedlings every 20 days with the relevant amount of water of the corresponding month (50% of monthly precipitation height when irrigation occurred twice a month, or 100% of monthly precipitation height when irrigation occurred once a month).
2. Irrigation scheme A2 (frequent): irrigation of seedlings every seven days with 25% of the simulated monthly precipitation height.

One seedling per family and per irrigation scheme was included in the experiment. Seedlings representing population D1 were abnormal in a high ratio (66%), producing finally a limited number of viable normal seedlings that were not enough to represent D1 in both irrigation schemes. For this reason, D1 was excluded from the experiment in the growing chamber and the viable normal seedlings from this population were used only in the common garden test under field conditions.

The growth chamber experiment lasted for 3 years (2013–2016). Measurements included seedling height, survival and leaf phenological traits (Madsen, 1994; Minotta and Pinzauti, 1996; Hiura, 1998; Whiteley et al., 2003; Kanaga et al., 2008). Seedling survival was monitored every week and the non-surviving ones were removed. Seedling height was measured at the end of each growing season from October 2014 until October 2016. Annual height growth between the experimental years 2014–2015 and 2015–2016 was also calculated. Phenology measurements included bud burst (leaf emergence), leaf senescence and duration of the growing period. Both parameters were expressed in number of days. The beginning of bud burst was considered to occur when the bud scales opened in a way that the newly emerged leaves were visible. Individual plants were considered entering the senescence stage when at least 50% of their leaves were discolored (Gömöry et al., 2015). Measurements of phenological traits were conducted in 3 day–intervals. The plastic response between subsequent experimental years was expressed for all traits as the difference of the annual mean values between these years.

### Provenance Experiment Under Field Conditions

A common environment provenance test under natural field conditions was set up in Agios Georgios (Drama, Greece), the same location that was used as a reference for the climate simulation in the growth chamber. An area of 100 m^2^ was fenced and a total number of 480 seedlings (two seedlings/family/population) were planted in a natural beech stand with dense crown closure (70–80%). Seedlings were planted with the soil substrate of their original pots (4 turf/2 sand/1 perlite). Survival measurements were taken two times per year during the months of October and April for 3 years. The survival percentage at the end of the 3 years experiment was used in this study. This experimental site was established in order to provide a direct comparison of seedling survival between the simulated future conditions applied in the growth chamber and the current natural forest conditions. Due to the absence of meteorological stations in the broader area and the lack of ecological studies for the specific site, the local environmental conditions were not described in detail and they were not monitored throughout the experiment.

### Basic Statistical Analysis

A comparison of means among populations and between provenances, was performed for the variables measured in the seedlings growing in the growth chamber and in the field. Differences in seedling survival among populations were described through a repeated pairwise chi-square test, since survival was scored as a binary variable. The same test was also used to evaluate the differences between the irrigation schemes, separately for each population. Mean differences in seedling height and annual growth were tested for significance using the analysis of variance (ANOVA) and the LSD criterion, since normal distribution and homoscedasticity of data were proven. This comparison was performed separately for each irrigation scheme and year of the study. Pairwise differences in all phenological traits between populations for each irrigation scheme and year were tested for significance, using the non-parametric Mann-Whitney U test, since normal distribution assumptions were not met. For all comparisons, the software STATISTICA v.10 (STATSOFT inc) was used.

### Hierarchical Linear Multilevel Models (HLM)

To assess the effect of provenance, population and irrigation scheme on the dependent variables (all traits used in this study), we utilized a three-level hierarchical linear modeling approach (HLM; Raudenbush and Bryk, 2002), that considers the nested structure of the data in this study. The implementation of this modeling approach is standard in a variety of disciplines (Afshartous and Wolf, 2007) with varying terminology depending on discipline (the hierarchical model is also known as the mixed-effects model, the random-coefficient model, and in the context of panel data, the repeated-measures or growth-curve model). A major advantage in this type of model over the standard regression models, is the within group and between groups comparison and the improved accuracy of point estimates in model parameters (e.g., Katahira, 2016). The framework of the model considers *n*_*ijk*_ responses on the dependent variables, nested within the populations (*i* = 1, 2, …, 8) which are again nested within the provenances of Evros and Drama (*j* = 1, 2). The last level of this nested modeling structure is completed with the inclusion of the two different irrigation schemes (*k* = 1, 2). The first level of the model is described as:

(1) $$\begin{array}{l} {\text{y}}_{\text{i}j\text{k}}=\beta_{0ij\text{k}}+\beta_{1ij\text{k}}\times {\text{X}}_{\text{i}j\text{k}}+{\text{e}}_{\text{i}j\text{k}} \end{array}$$

where **y**_**ijk**_ is the trait as a continuous response variable, **X**_**ijk**_ denotes the level-1 predictor variable of populations nested within each provenance and ${\text{e}}_{\text{i}j\text{k}}\sim \text{N}\left(0,\sigma_{\text{e}}^{2}\right)$ is the observation-level deviation which is normally distributed. The β - coefficients of the slope in equation (1) are subsequently used as a response variable (second level):

(2) $$\begin{array}{l} \beta_{1ij\text{k}}={\text{b}}_{0j\text{k}}+{\text{b}}_{1j\text{k}}\times {\text{X}}_{\text{j}\text{k}}+{\text{r}}_{\text{j}\text{k}} \end{array}$$

where **X**_**jk**_ is the level-2 predictor factor (provenance) and ${\text{r}}_{\text{j}\text{k}}\sim \text{N}\left(0,\sigma_{\text{r}}^{2}\right)$ expresses the normally-distributed deviations at the provenance level. Finally:

(3) $$\begin{array}{l} {\text{b}}_{1j\text{k}}=\gamma_{0\text{k}}+\gamma_{1\text{k}}\times {\text{X}}_{\text{k}}+{\text{u}}_{\text{k}} \end{array}$$

where ***X_k_*** denotes the irrigation scheme factor and $u_{k}\sim N(0,\sigma_{u}^{2})$.

Variables “population,” “provenance,” and “irrigation scheme” were the fixed effects in our models, whereas random effects terms were the corresponding errors not explained by the three fixed effects, i.e., **e**_**ijk**_, **r**_**jk**_ and **u**_**k**_. For the provenance factors, “Evros” was used as a reference category, while the first population within each provenance was used as a reference category for the population factors. Finally, we have used the irrigation scheme A1 as a reference category of the irrigation scheme.

Acknowledging the universal principle that no true model exists (Box and Draper, 1987), we choose not to fit a unique model but instead fit several candidate (nested) models in terms of varying the number of covariates included as predictors, in order to select a final optimal model among these candidates, based on appropriately balancing goodness-of-fit with simplicity and utilizing the appropriate evaluation criterion. We did not opt for stepwise (either forward/backward) elimination methods for variable selection since the latter have been recognized to suffer from significant drawbacks (Hurvich and Tsai, 1990; Roecker, 1991). Hence, model fit was assessed by initially fitting the null model (Model 1), which includes only the grand mean as predictor. One new predictor variable is added for each subsequent model. While Model 1 includes only the intercept, Model 2 introduces the factor of the populations and Model 3 adds the component of provenances. Finally, Model 4 additionally includes the factor of irrigation scheme and essentially corresponds to the full model presented in equations (1–3). Every next model is compared in terms of fit performance with the previous one. The overall significance of each model is then evaluated through the likelihood ratio statistic (LRT) (based on the likelihood of each model), which acts as a stand-alone measure of goodness-of-fit, as well as through the model comparisons. The LRT is only valid if used to compare hierarchically nested models, as applied in our research. LRT is calculated through the following likelihood ratio statistic:

(4) $$\begin{array}{l} D=2\times \left(ln\left(likelihood_{M_{i}}\right)-ln\left(likelihood_{M_{i-1}}\right)\right) \end{array}$$

where M_i−1_ denotes the reduced model and M_i_ the model with the additional parameter. The lme4 library (Bates et al., 2012) of the R software was used to fit the HLM models.

## Results

### Seedling Survival

**Figure 3:**
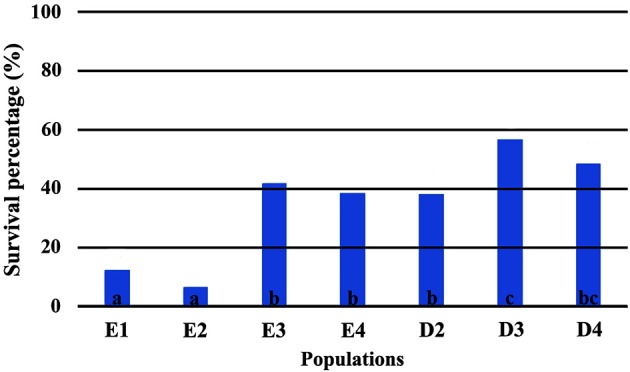
Survival percentages in the field*. *Values followed by the same letter do not differ among populations at 0.05 level of significance.

**Figure 4:**
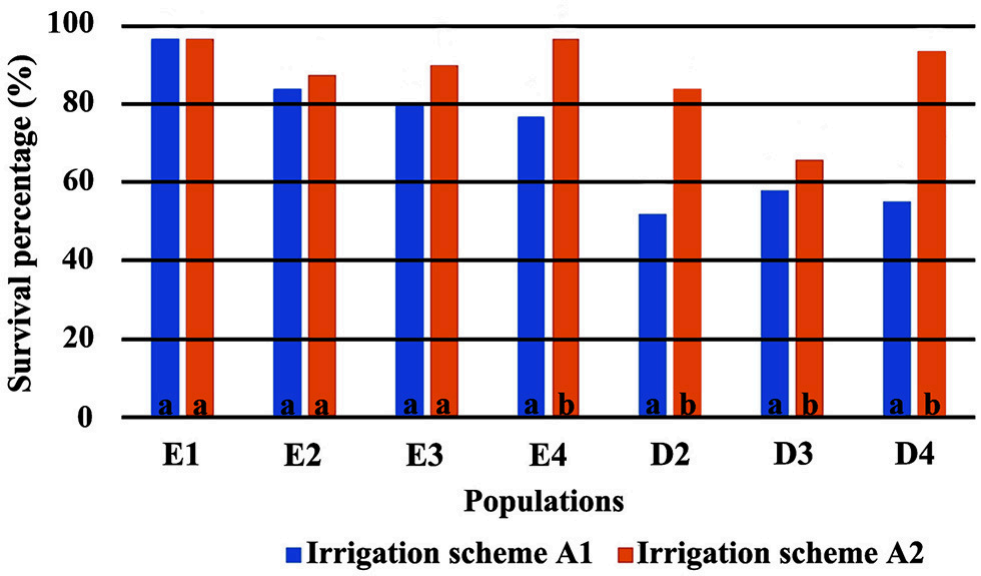
Survival percentages in the growth chamber *. *Values followed by the same letter do not differ between irrigation schemes for each population separately.

The direct comparison of survival percentages and the HLM model comparison produced similar results as far as seedling survival is concerned. The lowest survival rates were observed under field conditions for all populations, in comparison with the ones observed in the growth chamber (Figures 3, 4). Populations E1 and E2 showed the lowest and population D3 and the local population D4 the highest survival in the field (Figure 3). In the growth chamber, average survival was generally higher under frequent irrigation (scheme A2) than under less frequent irrigation (scheme A1). Besides E1 that demonstrated equal survival for both irrigation schemes, all populations showed lower survival percentages under A1 (Figure 4). A clear provenance pattern was observed in the chamber, with populations from Evros surviving better under longer drought intervals (A1 scheme) (Figure 4 and Table 1). No significant differences in seedling survival were found among populations under short drought intervals (scheme A2) (Table 1).

**Table 1:** Significance of differences (*p*-values) in final seedling survival among populations under irrigation schemes.

**Population**	**E1**	**E2**	**E3**	**E4**	**D2**	**D3**	**D4**
**IRRIGATION SCHEME A1**							
E1	–						
E2	ns	–					
E3	0.047	ns	–				
E4	0.024	ns	ns	–			
D2	0.000	0.010	0.029	ns	–		
D3	0.000	0.027	ns	ns	ns	–	
D4	0.000	0.016	0.044	ns	ns	ns	–
**IRRIGATION SCHEME A2**							
E1	–						
E2	ns	–					
E3	ns	ns	–				
E4	ns	ns	ns	–			
D2	ns	ns	ns	ns	–		
D3	ns	ns	ns	ns	ns	–	
D4	ns	ns	ns	ns	ns	ns	–

*ns, non significant*.

**Figure 5:**
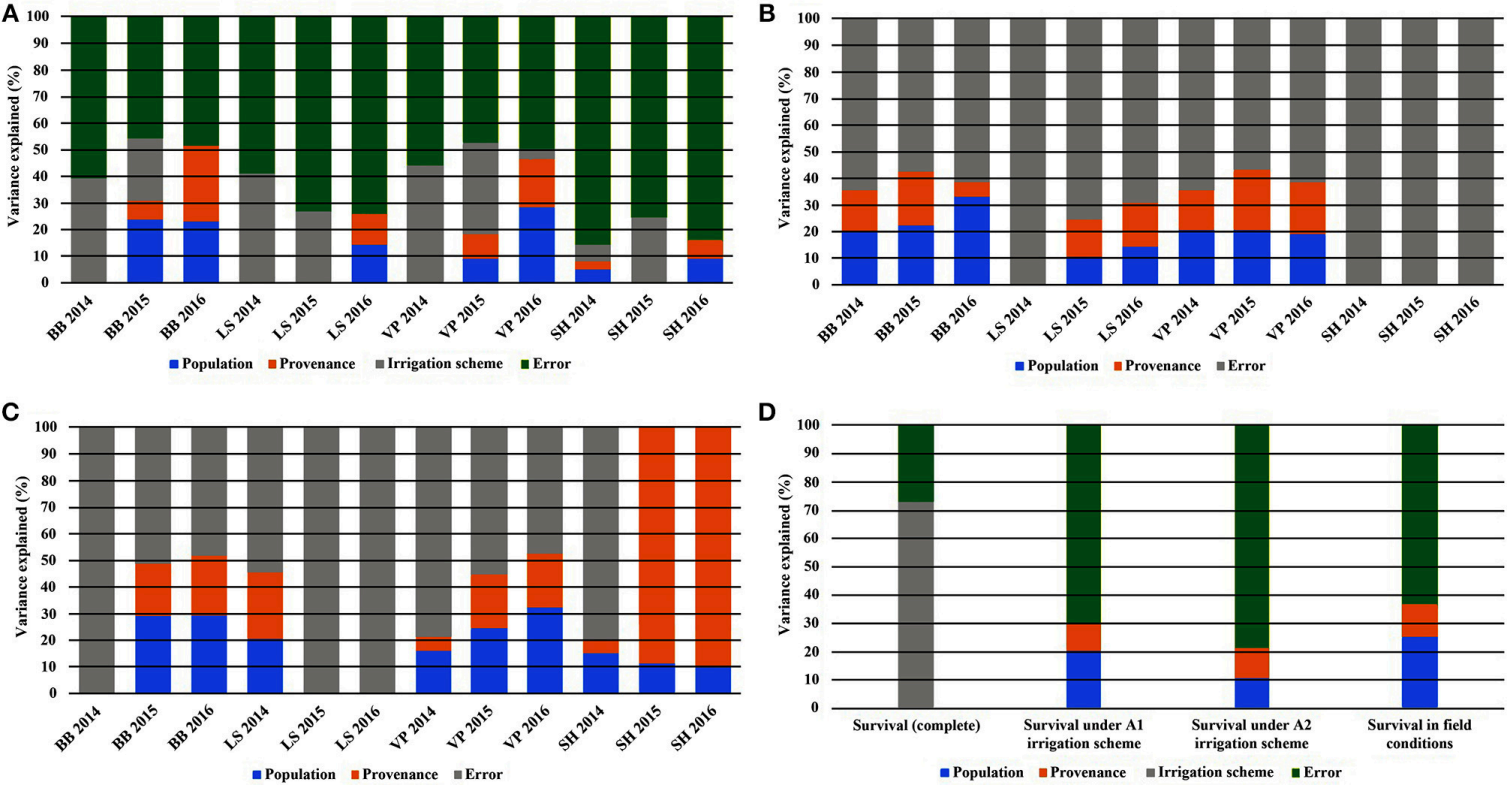
Partitioning of variance across the years of the study for the traits of leaf phenology* and seedling height overall **(A)**, under irrigation scheme A1 **(B)**, under irrigation scheme A2 **(C)**, and for seedling survival **(D)**. *Abbreviations: BB, Bud burst; LS, Leaf senescence; VP, Growing period; SH, Seedling height.

When the results of both experiments were considered in total, the comparison of the HLM models showed that only the growing environment had a significant effect on seedling survival (Tables 2, 3). Separately for irrigation scheme A1 in the growth chamber, provenance demonstrated a significant effect on survival, while no effect was recorded under A2. In the field experiment, population was the only significant factor affecting seedling survival. Accordingly, the partition of variance for the overall survival in both experiments was explained to a great extent by the different growing environments (73%), while provenance and population had no effect at all (Figure 5D). A larger proportion of variance was explained by population than by provenance under field conditions and under the irrigation scheme A1 in the growth chamber, while under A2 both provenance and population accounted for a small fraction of the overall variance (10.8 and 10.3%, respectively).

**Table 2:** Model comparisons of the HLM models for the survival data via LRT (D: likelihood ratio statistic; P: p-value of the statistical significance of LRT test).

**Dependent variables**	**Model 1 (null model)**	**Model 2 (population effects)**		**Model 3 (model 2 + provenance effects)**		**Model 4 (model 3 + irrigation scheme effects)**	
	***D***	***D***	***P***	***D***	***P***	***D***	***P***
Survival (complete)	492.86	487.04	ns	485.17	ns	469.67	0.000
Survival under A1 scheme	182.39	178.73	ns	167.20	0.000		
Survival under A2 scheme	69.77	61.23	ns	59.41	ns		
Survival in field conditions	183.72	165.37	0.005	162.13	ns		

**Table 3:** Parameter estimates for the best selected models (5% level of significance) for survival data.

**Independent variables**	**Dependent variables**			
	**Survival (complete)**	**Survival under A1 scheme**	**Survival under A2 scheme**	**Survival in field conditions**
**Intercept (**_**β_0 i j k_**_**)**	0.76	0.96	0.88	0.23
**Plot in Evros** (Ref: E1)				
E2	ns	ns		ns
E3	ns	ns		0.15
E4	n.s.	ns		0.16
**Plot in Drama** (Ref: D2)				**Plot in Drama** (Ref: D1)
D2	–	–		ns
D3	ns	ns		0.32
D4	ns	ns		0.26
**Provenance (Ref: Drama)**				
Evros	ns	0.41		
**Irrigation (ref: Irrigation scheme A1)**				
Irrigation scheme A2	0.14			
Irrigation G	−0.38			

*ns, non significant*.

### Seedling Height and Growth

**Figure 6:**
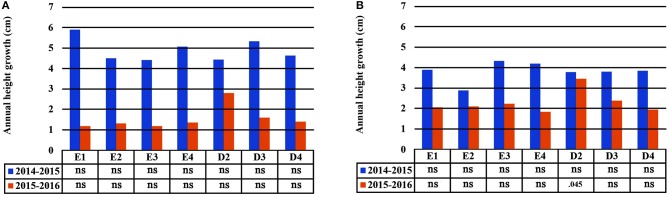
Plastic response of populations in seedling height between consecutive years of the experiment under irrigation schemes A1 **(A)** and A2 **(B)**.

In the growth chamber, seedlings from most populations reached a greater height under longer drought intervals (scheme A1), but this trend was statistically significant only for populations E1 and D3 during the first 2 years of the study (2014 and 2015) and for E3 in the first year (2014) only (Table 4 and Supplement Table 3). All differences between irrigation schemes A1 and A2 observed in 2014 and 2015 disappeared in 2016. Height increment between years 2015–2016 was almost halved in comparison to years 2014–2015 (Figures 6A,B). Significant differences in seedling height among populations were found only under irrigation scheme A2 during years 2014 and 2015 but not in 2016 (Table 4).

**Table 4:** Total seedling height at the end of each growth period under irrigation schemes A1 and A2 (Mean values±SE).

**Population**	Seedling height (cm)^*^					
	**2014**		**2015**		**2016**	
	**A1**	**A2**	**A1**	**A2**	**A1**	**A2**
E1	10.71 ^**A,a**^ (±0.48)	9.00 ^**A,b**^ (±0.41)	15.52 ^**A,a**^ (±0.79)	12.87 ^**AB,b**^ (±0.68)	17.27 ^A,a^ (±0.92)	15.42 ^A,a^ (±0.62)
E2	10.27 ^**A,a**^ (±0.61)	9.63 ^**AB,a**^ (±0.72)	14.59 ^**A,a**^ (±0.63)	12.85 ^**AB,a**^ (±1.14)	15.81 ^A,a^ (±1.08)	14.32 ^A,a^ (±0.68)
E3	12.13 ^**A,a**^ (±0.60)	10.73 ^**AB,b**^ (±0.27)	15.46 ^**A,a**^ (±0.80)	14.75 ^**B,a**^ (±0.94)	17.56 ^A,a^ (±0.98)	16.92 ^A,a^ (±0.74)
E4	10.70 ^**A,a**^ (±0.58)	11.08 ^**B,a**^ (±0.53)	14.36 ^**A,a**^ (±0.83)	14.69 ^**B,a**^ (±0.76)	16.42 ^A,a^ (±0.52)	17.17 ^A,a^ (±0.73)
D2	11.19 ^**A,a**^ (±0.78)	10.44 ^**AB,a**^ (±0.72)	14.93 ^**A,a**^ (±1.27)	13.70 ^**AB,a**^ (±0.63)	17.92 ^A,a^ (±1.26)	17.08 ^A,a^ (±1.22)
D3	10.58 ^**A,a**^ (±0.99)	8.98 ^**A,b**^ (±0.94)	15.06 ^**A,a**^ (±1.01)	12.02 ^**A,b**^ (±0.85)	17.50 ^A,a^ (±2.13)	15.83 ^A,a^ (±1.56)
D4	10.92 ^**A,a**^ (±1.03)	10.60 ^**AB,a**^ (±0.55)	14.90 ^**A,a**^ (±1.46)	13.41 ^**AB,a**^ (±0.72)	16.06 ^A,a^ (±1.31)	16.55 ^A,a^ (±0.83)

* *Values within columns followed by the same capital letter do not differ among populations for each irrigation scheme and year of study. Values within rows followed by the same small letter do not differ between irrigation schemes per population for each year of study separately*.

Seedling height in the growth chamber was not influenced significantly by population or provenance, according to the HLM model comparison. A significant influence of provenance was detected for height increment between 2015 and 2016 (Table 5), since provenance Drama demonstrated lower height growth than Evros during the same period (Supplement Table 3). The irrigation scheme was a factor that significantly influenced seedling height in 2015 and growth between 2014–2015 and 2015–2016 (Table 5). A large proportion of the total variance in height and growth traits was explained by the irrigation schemes after the first (6.5%) and even more so after the second year (24.4%), but not after the third year of the study. Provenances and populations explained smaller proportions of the total variance in 2014 (3.0 and 5.0%, respectively), had no effect in 2015 and explained a higher proportion of the phenotypic variance in seedling height in 2016 (6.6 and 9.3%, respectively). Provenance and population had no influence on seedling height increment between 2014–2015 but accounted for a larger fraction of variance in height increment between 2015–2016 (10.1 and 15.0%, respectively) (Figures 5A–C).

**Table 5:** Model comparisons of the HLM models for the complete data via LRT (D: likelihood ratio statistic; P: p-value of the statistical significance of LRT test).

**Dependent variables**	**Model 1 (null model)**	**Model 2 (population effects)**		**Model 3 (model 2 + provenance effects)**		**Model 4 (model 3 + irrigation scheme effects)**	
	***D***	***D***	***P***	***D***	***P***	***D***	***P***
**BB 2014**	1247.2	1242.2	ns	1240.7	ns	1232.5	0.004
**BB 2015**	1241.8	1212.8	0.000	1191.0	0.000	1183.9	0.000
**BB 2016**	1267.4	1230.3	0.000	1207.3	0.000	1204.2	ns
**LS 2014**	923.05	912.1	ns	900.56	0.000	893.7	0.008
**LS 2015**	1068.8	1060.9	ns	1059.2	ns	1052.2	0.008
**LS 2016**	1028.3	1014.7	0.018	1014.6	ns	1014	ns
**VP 2014**	1267.4	1261.5	ns	1261.5	ns	1252.7	0.003
**VP 2015**	1281.3	1256.3	0.000	1233.7	0.000	1225.2	0.000
**VP 2016**	1302.3	1270.0	0.000	1251.5	0.000	1248.1	ns
**SH 2014**	680.42	673.39	ns	673.24	ns	672.74	ns
**SH 2015**	771.03	763.74	ns	763.43	ns	758.45	0.025
**SH 2016**	772.97	766.08	ns	765.00	ns	762.97	ns
**GD 2014-20105**	690.65	687.61	ns	686.32	ns	681.12	0.022
**GD 2014-2015**	527.75	521.75	ns	506.35	0.000	500.70	0.017

*ns (non significant). Symbols: BB, Bud burst; LS, Leaf senescence; VP, Growing period; SH, Seedling height; GD, Height difference*.

### Leaf Phenological Traits

In the growth chamber, beech seedlings demonstrated longer growing periods in all populations and during all years under frequent irrigation (scheme A2), in comparison to scheme A1, where irrigation was less frequent and drought intervals were longer. In the spring of 2014, bud burst occurred significantly earlier in the growth chamber under irrigation scheme A2, in comparison to A1, for seedlings belonging to all populations besides E1 and E4 (Table 6). However, in 2015, only populations E2 and E3 continued to have significant differences in bud burst between A1 and A2, while in 2016, no significant differences could be observed between the two irrigation schemes. For leaf senescence, significant differences between the irrigation schemes were observed in all populations of Drama in 2014 and in some populations in 2015 (D3 and D4), but not in 2016 (Table 6). In all cases for which significant differences were observed, leaf senescence under A2 occurred later than under A1. Significant differences in the length of the growing period between A1 and A2 schemes were present for all populations besides E1 and E4 in 2014 (Table 6). In 2015, only population E3 did not differ significantly between A1 and A2 and finally in 2016 no difference between the two schemes was detected.

**Table 6:** Phenology of seedlings under irrigation schemes for the years of study (Mean values±SE).

**Population**	**2014**		**2015**		**2016**	
	**A1**	**A2**	**A1**	**A2**	**A1**	**A2**
BUD BURST DAYS^*^						
E1	101^A,a^ (±4.21)	98^A,a^ (±3.26)	120^A,a^ (±2.83)	111^A,a^ (±2.57)	120^AC,a^ (±2.94)	119^A,a^ (±4.60)
E2	114^BC,a^ (±3.90)	97^A,b^ (±2.64)	114^AB,a^ (±2.48)	103^AB,b^ (±4.01)	122^A,a^ (±2.72)	120^A,a^ (±2.28)
E3	117^BC,a^ (±4.71)	94^A,b^ (±4.22)	113^AC,a^ (±5.20)	100^AB,b^ (±5.33)	119^AC,a^ (±3.81)	119^A,a^ (±5.38)
E4	107^AB,a^ (±4.91)	100^A,b^ (±3.01)	119^A,a^ (±4.37)	111^A,a^ (±3.01)	123^A,a^ (±3.09)	120^A,a^ (±2.87)
D2	115^ABC,a^ (±3.27)	95^A,b^ (±2.24)	105^BCD,a^ (±6.07)	93^B,a^ (±2.24)	108^BC,a^ (±5.17)	97^B,a^ (±3.27)
D3	116^BC,a^ (±6.27)	99^A,b^ (±3.04)	96^D,a^ (±4.19)	92^B,a^ (±3.04)	101^B,a^ (±4.15)	96^B,a^ (±6.01)
D4	125^C,a^ (±1.71)	94^A,b^ (±2.09)	105^CD,a^ (±4.92)	94^B,a^ (±2.09)	110^AB,a^ (±5.82)	101^B,a^ (±4.50)
LEAF SENESCENCE DAYS^*^						
E1	293^A,a^ (±0.72)	295^AB,a^ (±1.45)	278^A,a^ (±1.22)	286^A,b^ (±2.36)	298^A,a^ (±2.44)	302^A,a^ (±2.35)
E2	294^A,a^ (±0.93)	293^A,a^ (±0.81)	285^A,a^ (±2.05)	290^A,a^ (±2.23)	303^A,a^ (±1.49)	302^A,a^ (±2.78)
E3	292^A,a^ (±0.10)	297^AB,a^ (±2.69)	286^A,a^ (±2.47)	288^A,a^ (±3.69)	307^B,a^ (±1.82)	300^A,a^ (±2.20)
E4	293^A,a^ (±0.70)	296^AB,a^ (±0.99)	282^A,a^ (±3.01)	288^A,a^ (±1.54)	302^A,a^ (±1.09)	302^A,a^ (±1.27)
D2	295^A,a^ (±2.18)	302^B,b^ (±1.62)	281^A,a^ (±3.00)	288^A,a^ (±2.26)	299^A,a^ (±1.35)	301^A,a^ (±1.90)
D3	292^A,a^ (±0.10)	304^B,b^ (±2.13)	280^A,a^ (±4.05)	288^A,b^ (±0.10)	304^AB,a^ (±4.30)	310^B,a^ (±0.10)
D4	294^A,a^ (±1.64)	299^AB,b^ (±1.80)	283^A,a^ (±2.26)	289^A,b^ (±1.83)	299^A,a^ (±1.07)	300^A,a^ (±1.33)
GROWTH PERIOD DAYS^*^						
E1	191^A,a^ (±4.24)	197^A,a^ (±3.23)	157^A,a^ (±3.15)	174^A,b^ (±3.70)	178^A,a^ (±4.25)	182^A,a^ (±4.05)
E2	179^BC,a^ (±3.71)	196^A,b^ (±2.87)	170^B,a^ (±2.88)	186^AC,b^ (±4.68)	180^A,a^ (±2.93)	182^A,a^ (±3.66)
E3	174^BC,a^ (±4.71)	202^AC,b^ (±5.75)	172^BC,a^ (±5.56)	186^AC,a^ (±6.17)	188^AB,a^ (±4.22)	181^A,a^ (±6.04)
E4	185^AB,a^ (±4.64)	194^AD,a^ (±3.37)	162^AB,a^ (±4.15)	177^A,b^ (±3.45)	179^A,a^ (±3.14)	182^A,a^ (±3.10)
D2	179^ABC,a^ (±4.70)	205^BC,b^ (±4.17)	175^BC,a^ (±7.49)	194^BC,b^ (±3.40)	191^AB,a^ (±6.11)	205^B,a^ (±4.18)
D3	175^BC,a^ (±6.27)	204^AB,b^ (±5.88)	184^C,a^ (±1.56)	195^BC,b^ (±3.04)	203^B,a^ (±8.10)	214^C,a^ (±6.01)
D4	168^C,a^ (±2.69)	204^BCD,b^ (±3.81)	177^BC,a^ (±6.01)	194^BC,b^ (±2.99)	189^AB,a^ (±5.87)	199^BC,a^ (±5.02)

* *Values of phenological traits within columns followed by the same capital letter do not differ among populations for each irrigation scheme and year of study. Values of phenological traits within rows that followed by the same small letter do not differ between irrigation schemes per population for each year of study separately*.

Differences in leaf phenological traits were also observed among populations within each irrigation scheme. Under longer drought intervals (scheme A1), differences in bud burst among populations were recorded in all years of the experiment, while such differences were observed in 2015 and 2016 only, when irrigation was more frequent (scheme A2) (Table 6). In the first year of the experiment (2014), under A1, population E1 had the earliest bud burst and D4 the latest. This pattern largely changed in 2015, with E1 and E4 demonstrating the latest bud burst, while all Drama populations showed the earliest bud burst. In both 2015 and 2016, seedlings belonging to provenance Evros demonstrated a delay in bud burst in comparison to provenance Drama, under both irrigation schemes.

For leaf senescence, differences among populations in 2014 were found only under frequent irrigation (A2 scheme), in contrast to bud burst. No difference was recorded in 2015 for both schemes, while minor differences among populations were recorded in 2016 (Table 6). Under longer drought intervals (irrigation scheme A1), differences among populations occurred in the third year of the experiment (2016), with seedlings belonging to population E3 demonstrating a later leaf senescence than the remaining populations. Under A2, leaf senescence occurred the earliest in population E2 and the latest in D2 and D3 during 2014, while in 2016 only population D3 showed a significantly later leaf senescence.

Populations demonstrated significant differences among each other in the duration of the growing period for both irrigation schemes and for all years (Table 6). Under less frequent irrigation (scheme A1), populations E1 and E4, contrary to E2 and E3, had the longest vegetation period in 2014, but the shortest in 2015 and 2016. On the contrary, under the same irrigation scheme, populations D3 and D4 had the shortest growing period in 2014, which gradually increased in 2015 and 2016, as compared to D2. Populations belonging to Drama provenance had a longer growing period during all years under frequent irrigation (scheme A2), especially during 2016.

**Figure 7:**
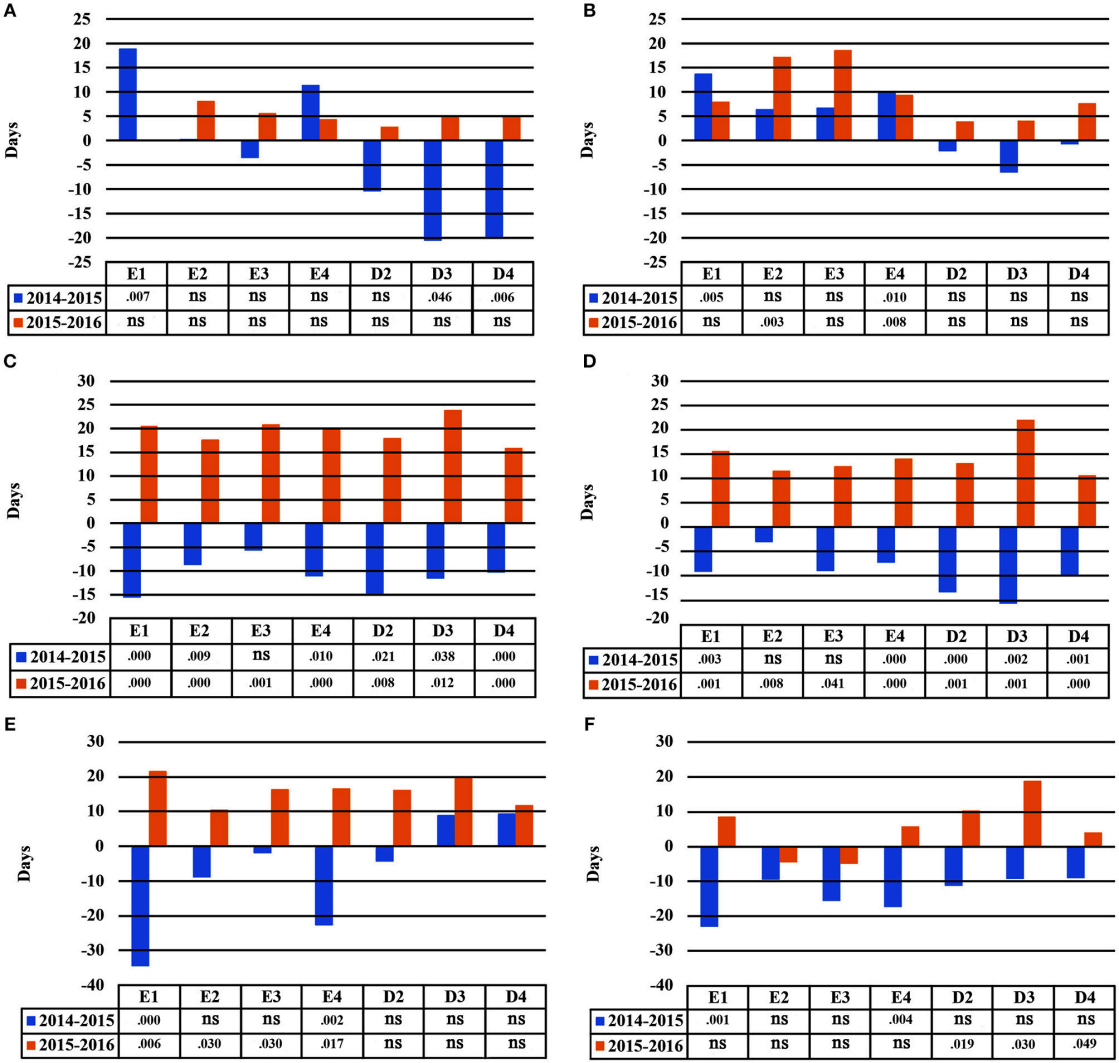
Plastic response of populations between consecutive years of the experiment under irrigation schemes A1 and A2 respectively for bud burst **(A,B)**, leaf senescence **(C,D)**, growing period **(E,F)**.

Seedlings demonstrated plastic responses between subsequent years in this study (Figure 7). The greatest delay in bud burst between years 2014–2015 was found for population E1 (+19 days), while seedlings belonging to D3 and D4 flushed much earlier in the second year of the study (−21 and −20 days, respectively), under A1. Under irrigation scheme A2, populations E2 and E4 had a significantly delayed bud burst in 2016 as compared to 2015. For leaf senescence, all populations showed a similar plastic response trend for both irrigation schemes A1 and A2 (Figure 7). Seedlings presented an earlier leaf senescence in 2015 as compared to 2014, and a later leaf senescence in 2016 as compared to 2015. However, results were more pronounced under scheme A1 and the populations showing the largest response were E1, D2 and D3. Finally, the two provenances (Evros-Drama) showed different plastic response trends for the length of the growing period. The largest plastic response was observed under A1, where the populations from Evros, especially E1 and E4, had a shorter growing period in 2014–2015 than in 2015–2016, while D3 and D4 had a longer one for the same year. In 2016, all populations demonstrated an increased growing period under A1. An opposite trend was observed under irrigation scheme A2, with all populations having a shorter growing period in 2015 and a longer one in 2016 (Figure 7).

The comparison of the HLM models revealed a similar trend for the timing of bud burst, leaf senescence and the length of the growing period, during the 3 years of the experiment (Table 5). The irrigation scheme had a significant influence on all traits in the first 2 years of the experiment (2014 and 2015), but not for the last year (2016). An opposite trend was observed for population and provenance that had significant effects on bud burst and growing period in 2015 and 2016, but not in 2014. Provenance showed a significant influence on the time of leaf senescence only in the first year and population only in the last year of the study. The frequency of irrigation (schemes A1 or A2) was the only fixed effect explaining a significant proportion of variance in 2014 for all traits, while in 2016, only (or mostly) provenance and population explained part of the variance. Provenance explained a larger proportion of variance (28.6%) than population (22.8%) for bud burst in the third year of the study, while an opposite trend was observed for leaf senescence (14.5% for population and 11.3% for provenance) and the growing period (28.3% for population and 18.6% for provenance).

## Discussion

### Seedling Survival and Height Growth

Seedling mortality in the growth chamber, for all populations and provenances, was observed exclusively during the first year of the experiment. In the environment of the growth chamber, irrigation frequency proved to be an important factor for seedling survival, since mortality increased when irrigation occurred after long intervals of drought (A1 scheme). In the field, the decisive factor for seedling survival proved to be the exposure to winter conditions. Frost events were not simulated in the growth chamber and seedlings survived better in the growth chamber than in the field, probably for this reason. This trend, where the largest differences in survival among populations occur when conditions are unfavorable, is common in provenance tests for beech (e.g., Banach et al., 2015).

Seedlings from Evros provenance generally presented higher survival in the growth chamber, under drought conditions, indicating a possible adaptation to prolonged drought intervals, since summer drought in Evros lasts longer and temperatures are generally higher than in Drama. Among the Evros populations, E4 presented significantly lower seedling survival under less frequent irrigation, indicating sensitivity to longer periods without rain. Indeed, population E4 is located in an area covered with beech forests characterized as an “island” of oceanic climate. This is emphasized by the frequent occurrence of *Galium rotundifolium*, a plant species acting as good indicator of oceanic climate (Ellenberg et al., 1992), which is absent in the Rodopi mountains, besides the area surrounding population E4. High variability in seedling survival and its response to irrigation frequency was also observed among populations within the Drama provenance, a possible indication of local adaptation of beech to the different microenvironments of each population.

In the field trial, survival results suggest the existence of a strong local adaptation pattern for beech seedlings, as other researchers also report (see Kreyling et al., 2012, 2014). The highest survival rate was observed for the two populations with a geographical origin closest to the location of the test site (D3 and D4). On the contrary, the more distant populations E1 and E2, that demonstrated the highest survival rates under drought conditions in the growth chamber, had the highest mortality rates under field conditions, in agreement with other beech common garden trials (Banach et al., 2015), although the opposite trend has been also reported (e.g., Sułkowska, 2004; Hofmann et al., 2015; Müller and Finkeldey, 2017). It is worth noting that adaptive differentiation to the colder conditions in the field experiment, as expressed in survival rates, was observed mainly among populations within provenances. Thus, seedlings from the distant Evros populations E3 and E4 survived as well as some populations of the local Drama provenance, indicating that environmental heterogeneity at a smaller geographical scale can create significant adaptive differentiation.

Under both irrigation schemes in the growth chamber and at the end of the third year all seedlings achieved similar heights without differences among populations and provenances. Similarly, Harter et al. (2015) did not find any differences in seedling height between two beech provenances under water deficit for 60 days. Various studies report that non-frequent irrigation leads to lower shoot height in seedlings (Arend et al., 2011; Thiel et al., 2014). However, in the current study, seedling height was generally larger under long drought intervals (irrigation scheme A1) rather than more frequent irrigation (scheme A2). In our study, the distribution pattern of water was more critical for seedling growth than the absolute amount of water received. It is possible that fewer but significant rain events may yield higher biomass increase than more frequent but minor events as it is already reported for plant communities (e.g., Bates et al., 2006; de Dios Miranda et al., 2010). This highlights the necessity for considering the rainfall patterns in terms of frequency and quantity rather than rainfall means alone as factor affecting the adaptation of plants (de Dios Miranda et al., 2009). Since this kind of irrigation pattern comparison has not yet been performed in any other common environment study for forest trees, the growth behavior of beech seedlings in this experiment needs to be further investigated using physiological and anatomical traits (Bolte et al., 2016). In any case, these responses to irrigation frequency seem to be temporary and reversible after a short time, revealing the existence of possible trade-offs between different traits as part of a complex adaptive strategy aiming at the best possible use of the available water.

### Leaf Phenological Traits

In our experiment, the two irrigation frequency schemes produced different leaf phenological responses at all traits during the first 2 years of the study. In the third year, the differences in leaf phenological traits were influenced mainly by provenance and population. Considering the adaptive significance of the timing of bud burst and leaf senescence, which define the length of the growing period, we assume that seedlings probably needed a certain period of time until they were able to physiologically adapt to the growth chamber conditions and irrigation schemes. The results of our study imply that the duration of the growing period in beech seedlings was mostly determined by the timing of bud burst, while the differences between populations, provenances, and irrigation schemes in leaf senescence were less pronounced. Bud burst occurred earlier and leaves matured later under frequent irrigation (scheme A2) especially during the first years of the study, a trend that was consistent for all populations. Thus, the existence of longer drought intervals between irrigation events (scheme A1) has shortened the growing period of plants in the chamber, an expected response to stressful conditions.

Bud burst is considered to be under strong genetic control (Dittmar and Elling, 2006; Doi et al., 2010; Abbott et al., 2015; Gömöry et al., 2015; Müller et al., 2017) and provenances often show adaptive differences in this trait in common environment experiments, that correspond to specific environmental conditions at the sites of origin (von Wühlisch et al., 1995; Schüler et al., 2012; Kramer et al., 2017). Provenance tests all over the continent describe a general trend of populations growing in warmer and less continental climates to have an earlier bud burst than populations from colder climates (Robson et al., 2011, 2018). Extrapolating this trend to the provenances and populations of our study, we would expect provenance Evros to have an earlier bud burst than Drama. While this was indeed the case in the first year of the study in the growth chamber, the phenological trend reversed during the next 2 years. Thus, in the first year of the growth chamber experiment, provenance Evros had an earlier bud burst and a longer growing period than Drama, while in the next years, bud burst had shifted to a later date for Evros and to an earlier date for Drama. These findings show that provenances initially demonstrated the expected genetic response matching the environmental conditions at their sites of origin, with Drama having a cooler and more temperate climate than Evros. However, the projected conditions of 2050 under climate change applied in the growth chamber, probably stimulated a differentiated plastic reaction for both provenances. We assume that the same environmental signals that trigger bud flush in plants, such as temperature and humidity, had a different effect on the provenances in this study. Indeed, in a temperature manipulation experiment, Fu et al. (2012) report that artificial warming of beech seedlings significantly affected bud burst date in different provenances.

Our findings reveal two different foliar phenology patterns followed by beech seedlings in this study. These patterns seem to depend on provenance and differ mainly in bud burst timing in spring and less significantly in the timing of leaf senescence in autumn. Seedlings from Evros provenance showed a delay in bud burst in 2015 and 2016 in respect to 2014. This pattern is apparent under both irrigation schemes, however it is more evident under A1, suggesting that Evros provenance is better adapted to prolonged intervals between irrigation events. On the other hand, populations of Drama showed earlier budburst in the respective years under both schemes. These observations reveal a different foliar phenology pattern, where beech populations initiate their growing period sooner in order to maximize total carbon gain, as it seems to be more prone to higher temperatures during summer, under non-frequent irrigation (scheme A1). Most reports from field provenance trials for beech show that, unlike in our study, bud burst timing remains constant and the order of provenances in this regard remains unchanged during years, without a strong interaction between provenance and test site (Robson et al., 2011, 2018). We assume that stressful environmental conditions, such as the ones simulated in the growth chamber in our study, may trigger certain physiological responses that will allow trees to survive. Since phenological traits are complex in nature and in their underlying mechanisms (Vitasse et al., 2010; Fu et al., 2012; Basler and Körner, 2014), further studies of provenances and families of trees utilizing functional and anatomical traits are needed to understand these adaptation strategies better.

### Adaptive and Phylogenetic Differentiation Patterns

The results of our study reveal the existence of high genetic diversity in adaptive traits in the beech forests of N.E. Greece. These adaptive differences occur at multiple spatial levels, among distant and neighboring populations. There is a clear geographical and environmental trend in adaptation to climate. On the eastern side of the study area (provenance Evros), beech populations are better adapted to dry climatic conditions with longer intervals of drought during the summer and low probability of late frosts in the spring. As a result, seedlings from Evros demonstrate higher survival and earlier bud burst in the first year of the study than seedlings from Drama, under simulated climate change conditions, especially when irrigation is not frequent. At the same time, most of the Evros populations show a specific phenotypic pattern, as a response to the simulated climate change conditions in the growth chamber, with a shortening of the growth period during the second year of the study. Respectively, populations located on the western side of the study area (provenance Drama) seem to demonstrate adaptations to more temperate conditions, characterized mainly by long and cold winters and more humid summers. Seedlings originating from Drama showed lower survival under long drought intervals during the summer in the growth chamber and a late bud burst in the first year, but then shifted their growing season earlier and flushed earlier than the Evros seedlings in the second year of the study.

Besides the different environmental conditions that may have caused adaptive genetic differentiation between the two provenances of this study, another reason for the differences in adaptive traits that exist between Evros and Drama may be the presence of different levels of a possible admixture between two beech species, *F. sylvatica* and *F. orientalis* that presumably form a contact zone in the southeastern part of Europe (e.g., Paule, 1995; Tsiripidis and Athanasiadis, 2003; Papageorgiou et al., 2008; Govaerts et al., 2013). In N.E, Greece, several authors suggest an increasing admixture trend toward the east (Moulopoulos, 1965; Tsiripidis and Athanasiadis, 2003; Papageorgiou et al., 2008; Hatziskakis et al., 2011), with provenance Evros being genetically and morphologically closer to *F. orientalis* and provenance Drama to *F. sylvatica*. Since the former species grows in warmer and drier climates than the latter, adaptive differentiation may exist between them, as suggested by earlier studies (Atalay, 1992; Tsiripidis and Athanasiadis, 2003; Papageorgiou et al., 2008).

Adaptive differences were recorded within provenances as well, indicating that beech populations that belong to the same geographic region and are located within a small distance exhibit large genetic differences in adaptive traits. Differences in altitude, aspect and topographical connectivity between populations probably define an environmental mosaic with semi-isolated patches of beech forests, where natural selection can locally cause well-adapted ecotypes that differ at a small spatial scale. In Evros, population E1 was proven to be best adapted to warmer conditions and summers with prolonged periods without rain, as shown by the high survival rate of E1 in the growth chamber, the early bud burst in the first year of the study and the defensive phenological plasticity pattern in the next years. Indeed, E1 shows adaptive differences from the neighboring population E2 (only 682 m apart), probably because the environmental conditions at these sites are critically different. Population E1 is a marginal beech stand located on a south facing slope, while E2 is a dense forest on the north facing slope of the mountain, growing under much more favorable conditions. Furthermore, population E4 showed a different adaptive pattern in seedling survival than E1, which can be attributed to the more oceanic microclimate of the specific location, as explained above.

Despite the differences in the survival rate between E1 and E4, these two populations demonstrated similarities in their phenological profile, which was much different than E2 and E3 in the Evros provenance. According to a fine scale genetic analysis of chloroplast DNA haplotypes in beech populations in the region (Manolis et al.: unpublished data), both E1 and E4 seem to derive from the postglacial expansion of the same beech population in a local refugium, where beech survived during multiple glacial cycles and probably developed effective adaptive strategies. The origin of a local glacial refugium has been suggested as a possible explanation for late bud burst of the Slovenian beech provenance Idrija-II/2 in European field trials, as a possible adaptation to long cold winters during glaciation (Brus, 2010; Robson et al., 2011, 2018). Thus, adaptive differences between distant or close-by populations may derive from selective evolutionary responses to environmental conditions of past refugia, in parallel with the ongoing processes of adaptation to current environmental conditions. This seems to be especially true in the case of disjunct “rear edge” populations of forest trees that have not received any maladaptive gene flow from the core populations of the species and maintain the ability to evolutionary adjust themselves to local climate changes (Fady et al., 2016).

### Conclusions

Both hypotheses tested in our study were confirmed. Beech seedlings deriving from populations of N.E. Greece were in general able to survive well under climate change conditions, simulated and applied in the growth chamber. Plants showed adaptive differences that allowed them to avoid high levels of mortality in the growth chamber. Furthermore, beech genotypes demonstrated phenological plastic responses to different environmental conditions and precipitation frequency in particular. Beech seedlings alter the duration of their growing season as a response to environmental signals, avoiding environmental stress and high selection pressure. In our study we were able to describe different adaptation strategies, that relate to the distribution patterns of specific environmental factors, rather than the average annual or monthly values of these measures. Indeed, fluctuations in temperature and precipitation within each year seem to be crucial for survival and growth, as well as the duration of the growing season. For this reason, provenance Evros is considered to be well-adapted to a less temperate climate, due to the low rainfall during summer, despite the high annual precipitation that occurs mainly during the winter in this region. Furthermore, our study proved the adaptive significance of the distribution of precipitation at a small temporal scale, since different adaptive strategies appeared among beech seedlings when the same amount of water was distributed differently within each month. This indicates that the physiological response mechanisms of beech individuals are very complex and depend on several interacting parameters that are difficult to study in total. For this reason, conclusions about the suitability of provenances for translocation and use in afforestation or reforestation projects should consider the small scale ecotypic diversity of the species and view multiple environmental and climatic parameters in connection to each other.

Despite the existence of adaptive diversity among the populations of beech in N.E. Greece, the survival of beech and other temperate forest tree species in the future remains unknown, since the speed, the uniformity and the intensity of climate change are different in different climate models. We expect severe climate fluctuation in the near future, with an increased intensity in the forests of the Mediterranean ecoregion being most at risk. Beech populations in the rear edge of the distribution of the species have a large adaptive potential and their persistence seems to be of major importance for forests and forestry all over Europe, pressing for an adjustment of forest management and conservation policies (Mátyás et al., 2009; Lefèvre et al., 2014; Fady et al., 2016).

## Data Availability Statement

The datasets analyzed for this study can be found in ResearchGate [www.researchgate.net]. doi: 10.13140/RG.2.2.15294.13128; doi: 10.13140/RG.2.2.11099.82727.

## Author Contributions

GV, AP, and OG developed the original idea of the research, GV, AP, TM, and ITa planned the experiments in the chamber and the field, GV, AM, and ITs planned sampling and collected the seed, GV, TM, AM, and ITa supervised the experiment in the growth chambers, GV, AP, OG, and ITs analyzed the data, CM and AP developed the models, GV developed the first draft of the manuscript and all authors contributed in preparing the manuscript in its final form.

### Conflict of Interest Statement

The authors declare that the research was conducted in the absence of any commercial or financial relationships that could be construed as a potential conflict of interest.
