# Supplementary material for: Adaptive Diversity of Beech Seedlings Under Climate Change Scenarios
Source: Front Plant Sci. 2019 Jan 8;9:1918. doi: 10.3389/fpls.2018.01918 (PMC6331410; doi:10.3389/fpls.2018.01918)
Supplement: Supplementary file 2 [file Table_2.DOCX]

**Supplement Table 2.** Climatic parameters derived from CSIRO MK3 CGM and SRES A1B for the reference area.

| **Month** | **Mean monthly temperature (^o^C)** | **Mean monthly minimum temperature (^o^C)** | **Mean maximum monthly temperature (^o^C)** | **Mean monthly precipitation (mm)** |
| --- | --- | --- | --- | --- |
| January | 0.2 | -3.2 | 3.5 | 57.6 |
| February | 1.7 | -2.1 | 5.5 | 51.8 |
| March | 4.0 | -0.4 | 8.4 | 48.2 |
| April | 8.8 | 3.6 | 13.9 | 45.8 |
| May | 13.7 | 8.1 | 19.2 | 62.6 |
| June | 18.2 | 12.2 | 24.0 | 53.6 |
| July | 21.7 | 15.4 | 28.0 | 32.8 |
| August | 21.6 | 15.5 | 27.8 | 26.0 |
| September | 17.5 | 11.6 | 23.4 | 29.2 |
| October | 11.6 | 6.2 | 16.8 | 36.0 |
| November | 6.0 | 1.9 | 10.1 | 56.2 |
| December | 2.4 | -1.1 | 5.9 | 67.4 |
| **Mean value** | 10.6 | 5.6 | 15.5 | 47.2 |
